# Sequencing and Analysis of Complete Chloroplast Genomes Provide Insight into the Evolution and Phylogeny of Chinese Kale (*Brassica oleracea* var. *alboglabra*)

**DOI:** 10.3390/ijms241210287

**Published:** 2023-06-17

**Authors:** Yilin Wang, Qiannan Liang, Chenlu Zhang, Huanhuan Huang, Hao He, Mengyu Wang, Mengyao Li, Zhi Huang, Yi Tang, Qing Chen, Huiying Miao, Huanxiu Li, Fen Zhang, Qiaomei Wang, Bo Sun

**Affiliations:** 1 College of Horticulture, Sichuan Agricultural University, Chengdu 611130, China; 2021205033@stu.sicau.edu.cn (Y.W.); 2022205029@stu.sicau.edu.cn (Q.L.); 2022205036@stu.sicau.edu.cn (C.Z.); hh820423@163.com (H.H.); 202001670@stu.sicau.edu.cn (H.H.); limy@sicau.edu.cn (M.L.); huangzhi@sicau.edu.cn (Z.H.); 13920@sicau.edu.cn (Y.T.); supnovel@sicau.edu.cn (Q.C.); 10650@sicau.edu.cn (H.L.); zhangf@sicau.edu.cn (F.Z.); 2 Department of Horticulture, Zhejiang University, Hangzhou 310058, China; wangmengyu@zju.edu.cn (M.W.); miaoamiao@zju.edu.cn (H.M.)

**Keywords:** chloroplast genomes, genome assembly and annotation, microsatellites, inverted repeat, genome comparison, phylogeny

## Abstract

Chinese kale is a widely cultivated plant in the genus *Brassica* in the family Brassicaceae. The origin of *Brassica* has been studied extensively, but the origin of Chinese kale remains unclear. In contrast to *Brassica oleracea*, which originated in the Mediterranean region, Chinese kale originated in southern China. The chloroplast genome is often used for phylogenetic analysis because of its high conservatism. Fifteen pairs of universal primers were used to amplify the chloroplast genomes of white-flower Chinese kale (*Brassica oleracea* var. *alboglabra* cv. Sijicutiao (SJCT)) and yellow-flower Chinese kale (*Brassica oleracea* var. *alboglabra* cv. Fuzhouhuanghua (FZHH)) via PCR. The lengths of the chloroplast genomes were 153,365 bp (SJCT) and 153,420 bp (FZHH) and both contained 87 protein-coding genes and eight rRNA genes. There were 36 tRNA genes in SJCT and 35 tRNA genes in FZHH. The chloroplast genomes of both Chinese kale varieties, along with eight other Brassicaceae, were analyzed. Simple sequence repeats, long repeats, and variable regions of DNA barcodes were identified. An analysis of inverted repeat boundaries, relative synonymous codon usage, and synteny revealed high similarity among the ten species, albeit the slight differences that were observed. The Ka/Ks ratios and phylogenetic analysis suggest that Chinese kale is a variant of *B. oleracea*. The phylogenetic tree shows that both Chinese kale varieties and *B. oleracea* var. *oleracea* were clustered in a single group. The results of this study suggest that white and yellow flower Chinese kale comprise a monophyletic group and that their differences in flower color arose late in the process of artificial cultivation. Our results also provide data that will aid future research on genetics, evolution, and germplasm resources of Brassicaceae.

## 1. Introduction

Chloroplasts are intracellular organelles in plants that contain all the elements required for photosynthesis, as well as the biosynthesis of amino acids, nucleotides, lipids, and starch [1]. The chloroplast genome is one of the three major genetic systems of plants; it exists in a single copy and is maternally inherited. Recombination of the chloroplast genome is rare; the chloroplast genome is also not affected by gene deletion, overlap, or pseudogenes. Therefore, chloroplast genomes have been used as DNA barcodes for the identification of plants and in phylogenetic analyses [2]. The chloroplast genome in most terrestrial plants and some algae has a highly conserved tetrad structure. It comprises one large single-copy (LSC) region of approximately 81–90 kb, one small single-copy (SSC) region of approximately 18–20 kb, and two inverted repeat (IR) sequences of approximately 20–30 kb [3]. The chloroplast genome is usually 120–160 kb and contains 100–131 genes [4,5]; genes in the chloroplast genome play roles in photosynthesis, transcription, translation, and biosynthesis [6]. The abundance of chloroplast genes with different functions varies among plants, and this is associated with the adaptation of plants to environmental changes. Owing to the small size of the chloroplast genome, as well as its conserved structure and gene content, chloroplast genomes have often been used in genetic and evolutionary studies of plants [7].

Chinese kale (*Brassica. oleracea* var. *alboglabra*) is an annual or biennial herb native to south China. *B. oleracea* usually has yellow flowers. Bailey was the first to investigate the taxonomy of Chinese kale, and he designated white-flower Chinese kale as a species named *B. alboglabra* [8]. Sinskaia found that Chinese kale can easily hybridize with heading *B. oleracea*, and he believed that Chinese kale is a variant of *B. oleracea* [9]. The chromosome structure and pollen morphology of Chinese kale and *B. oleracea* var. *oleracea* are similar [10], which suggests that Chinese kale is a variant of *B. oleracea* [11,12,13].

*B. oleracea* is thought to have originated from the Mediterranean coast [14]; it is one of the six cultivated *Brassica* species in the Triangle of U [15]. There are several variants of *B. oleracea*, such as *B. oleracea* var. *capitata*, *B. oleracea* var. *botrytis*, *B. oleracea* var. *italica*, *B. oleracea* var. *gemmifera*, *B. oleracea* var. *acephala*, *B. oleracea* var. *caularapa*, and *B. oleracea* var. *sabauda*; Chinese kale (*B. oleracea* var. *alboglabra*) is also among these, and the relationship between them is complex due to their low reproductive isolation. *Sinapis alba*, also known as *B. hirta*, is a white-flowered or yellow-flowered annual plant in the family Brassicaceae [16].

Chinese kale with white flowers is the main cultivated species and it has a wide distribution. Chinese kale can also have yellow flowers, and this variant has been cultivated in China, mainly in Fujian and Taiwan. Therefore, we obtained the complete chloroplast genomes of Chinese kale and explored its taxonomic status.

In this study, the chloroplast genomes of Chinese kale with white and yellow flowers were sequenced using the Illumina HiSeq platform, and then assembled, annotated, and analyzed. We described the basic characteristics of these two chloroplast genomes, obtained candidate chloroplast molecular markers for identification, and examined the phylogenetic relationships among Chinese kale and their related species.

## 2. Results

### 2.1. Chloroplast Genome Characterization

We sequenced the chloroplast genomes of SJCT (*Brassica oleracea* var. *alboglabra* cv. Sijicutiao (SJCT)) and FZHH (*Brassica oleracea* var. *alboglabra* cv. Fuzhouhuanghua (FZHH)). Both of them possessed the typical quadripartite structure, with complete lengths of 153,365 bp and 153,420 bp, respectively. Both chloroplast genomes contain a pair of IRs (26,197 bp) and an SSC region (17,834 bp). SJCT contains an LSC region with a length of 83,137 bp; the LSC region in FZHH has a length of 83,192 bp. The GC content of the two chloroplast genomes was 35.44%.

**Figure 1:**
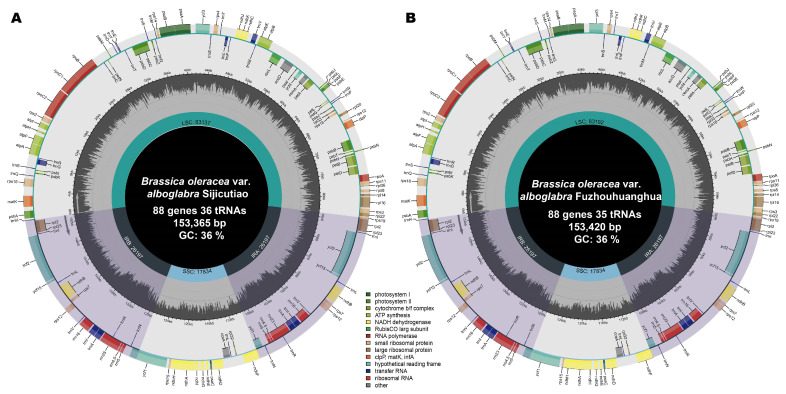
The complete chloroplast genome maps of (**A**) *B. oleracea* var. *alboglabra* cv. Sijicutiao (SJCT) and (**B**) *B. oleracea* var. *alboglabra* cv. Fuzhouhuanghua (FZHH). The genes in the circle are transcribed clockwise, and the genes outside are transcribed counterclockwise. Genes are color-coded according to their roles. The G/C content of each gene is shown in the deep grey in the central circle and the A/T content is shown in lighter grey.

There were 88 protein-coding genes and eight rRNA genes in both Chinese kale varieties; there were 36 tRNA genes in SJCT and 35 tRNA genes in FZHH. In SJCT, the protein-coding genes were divided into four groups according to their functions, with 45 genes related to photosynthesis, 74 genes related to self-replication, eight genes with unknown functions, and five other genes (Supplemental Tables S1 and S2). The chloroplast genome of FZHH was found to lack the *trnD* gene, which was caused by a fragment insertion in the middle of the *trnD* gene sequence in FZHH (Figure 1).

### 2.2. SSRs and Repeat Sequence Analysis

**Figure 2:**
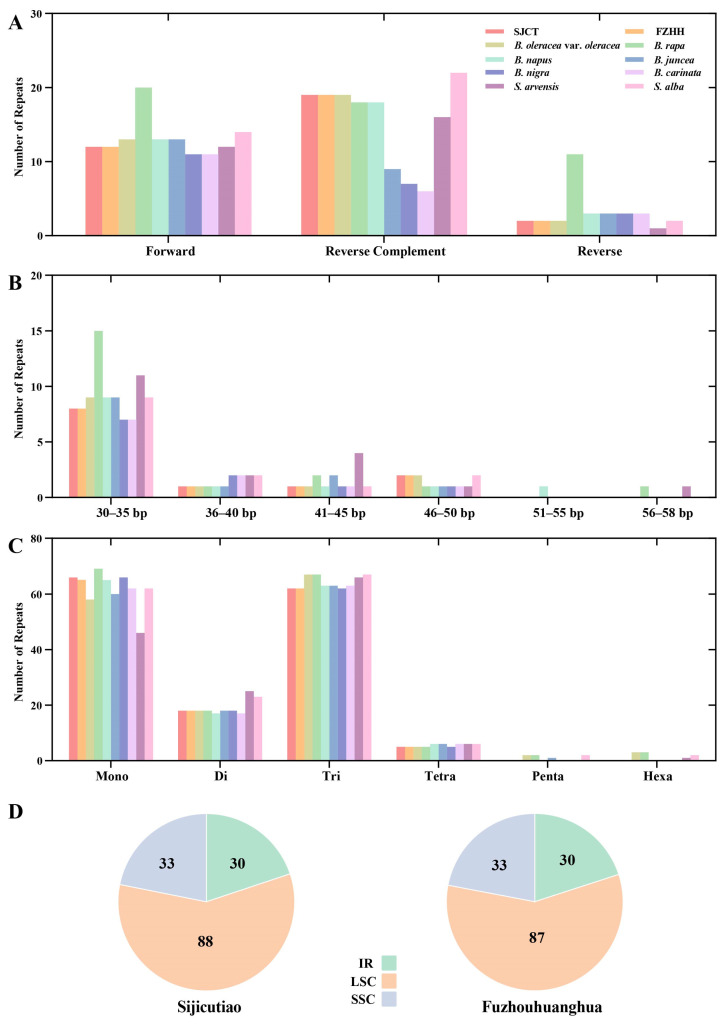
Dispersed repeated sequence analyses in 10 chloroplast genomes. (**A**) Number of three types of repeated sequences in 10 chloroplast genomes; (**B**) Number of dispersed repeated sequences with different period sizes; (**C**) Number of SSRs with different sizes; (**D**) Statistics of different SSR locations in SJCT and FZHH.

An analysis of the long repeats showed that both Chinese kale varieties contained 12 forward repeats, 19 reverse complement repeats, and two reverse repeats. In the chloroplast genomes of the two Chinese kale varieties, six *Brassica* and two *Sinapis*, there were 11–20 forward repeats and 6–22 reverse complement repeats. All the species had 2–3 reverse repeats, with the exception of *B. rapa*, which had the maximum number of reverse repeats (up to 11); most of these reverse repeats were caused by single-base duplications of A (Figure 2A). Among the forward repeats, there were eight repeats in both Chinese kale varieties with a size of 30–35 bp; one repeat was 37 bp, one repeat was 43 bp, and two repeats were 46 bp and 47 bp, respectively (Supplemental Table S3). In the ten chloroplast genomes, there were 7–15 forward repeats of 30–35 bp, 1–2 forward repeats of 36–40 bp, 1–4 forward repeats of 41–45 bp, and 1–2 forward repeats of 46–50 bp. (Figure 2B). In addition, *B. napus* has one repeat of 51–55 bp. *B. rapa* has an identical continuous 58-bp repeat located in the non-coding region between *atpH* and *atpI*. *S. arvensis* also has one repeat of 56–58 bp. In the chloroplast genome of SJCT, an analysis of SSRs revealed 66 mononucleotides, 18 dinucleotides, 62 trinucleotides, five tetranucleotides, and no pentanucleotides or hexanucleotides. FZHH contained one less mononucleotide than SJCT. In the 10 chloroplast genomes, 46–69 mononucleotides, 17–25 dinucleotides, 62–67 trinucleotides, 5–6 tetranucleotides, 0–2 pentanucleotides, and 0–3 hexanucleotides were identified (Figure 2C). In the genome of SJCT, there were 30, 88, and 33 SSRs identified in the IR, LSC, and SSC regions, respectively. In FZHH, 30, 87, and 33 SSRs were identified in the IR, LSC, and SSC regions, respectively (Figure 2D). Most of the SSRs located in the protein-coding genes were trinucleotides (Supplemental Table S3).

### 2.3. RSCU Analysis

**Figure 3:**
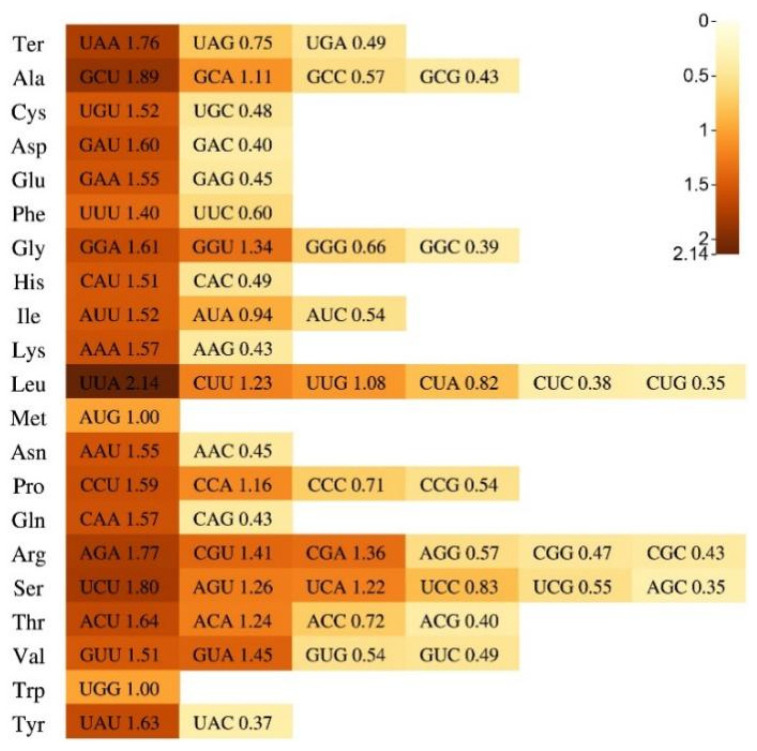
Amino acids and stop codons of RSCU for the protein-coding regions in Chinese kale.

A total of 23,063 (SJCT), 23,063 (FZHH), 23,102 (*B. nigra*), 23,233 (*B. carinata*), 23,100 (*B. rapa*), 23,636 (*B. napus*), 23,143 (*B. oleracea* var. *oleracea*), 21,383 (*B. juncea*), 26,658 (*S. arvensis*), and 26,081 (*S. alba*) protein-coding sequences were identified in each of the 10 species. The results of the RSCU analysis were similar (Supplemental Table S4). If RSCU > 1, this codon is considered an optimal codon with high preference. In Chinese kale, there were 32 optimal codons, and most of them ended with A/T. According to the results, the most frequent amino acid was UUA, which encodes leucine, and the least frequent amino acids were CUG and AGC (Figure 3).

### 2.4. Chloroplast Genome Comparison

**Figure 4:**
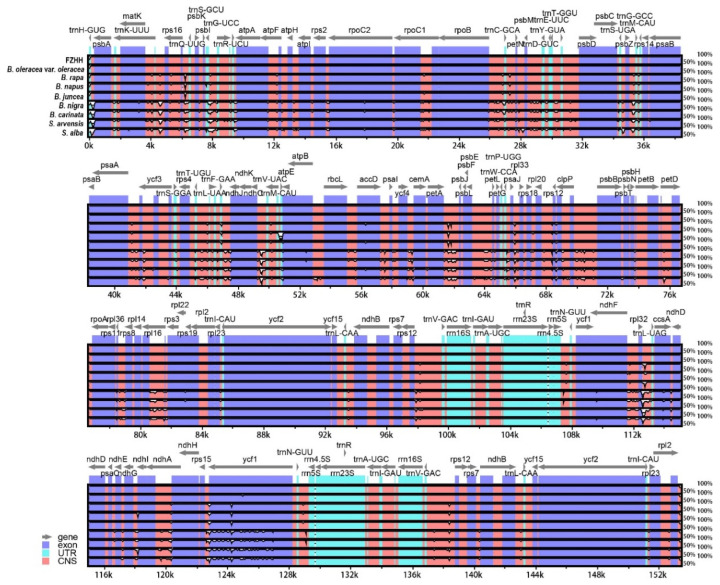
mVISTA-based visual representation of the aligned genomes of the ten chloroplast genomes using annotation of SJCT chloroplast genome as the reference.

**Figure 5:**
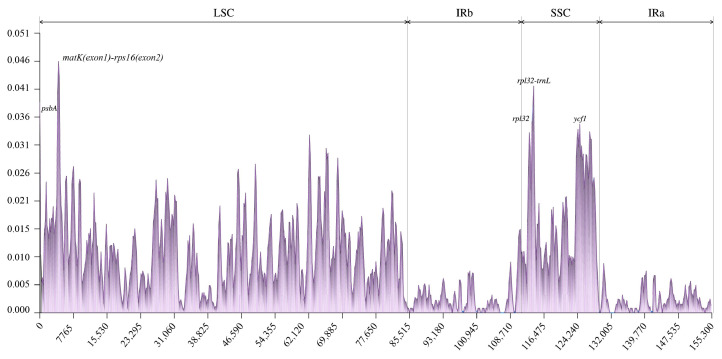
The nucleotide variability (Pi) values were compared among ten chloroplast genomes. Position of the window midpoint on the *X*-axis and the nucleotide diversity within each window on the *Y*-axis (window length: 600 bp, step size: 200 bp).

A comparison of the variation in the 10 chloroplast genomes using SJCT as a reference revealed a high degree of sequence similarity (Figure 4). This is consistent with the results of the mVISTA analysis, wherein the divergent hotspot regions indicated that the five mutational hotspots exhibited markedly higher π values. The *matK*-*rps16* showed the highest average sequence divergence among the 10 chloroplast genomes, followed by *rpl32*-*trnL* and *psbA* (Figure 5). These genes could be used for DNA barcoding research in the future. According to the chloroplast genome structure analysis, no genome rearrangements were detected across all 10 chloroplast genomes (Supplemental Figure S1).

**Figure 6:**
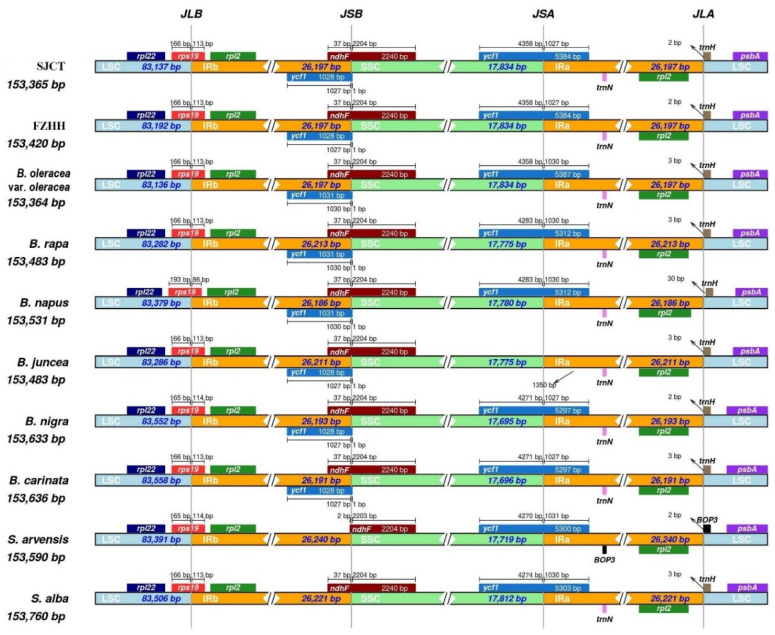
Comparison of LSC, IR, and SSC junction positions among ten chloroplast genomes.

The IR boundaries of SJCT and FZHH were similar to those of *B. oleracea* var. *oleracea* (Figure 6). All 10 chloroplast genomes were highly conserved. The gene *rps19* was detected at the LSC/IRb boundary, and there was only one base pair difference at this location across all the chloroplast genomes. Some differences were observed. First, in *B. juncea*, *ycf1* was lost at the SSC/IRa boundary, which was caused by the lack of an initiation codon. Second, in *S. arvensis*, the premature transcription termination of *ndhF* was observed at the IRb/SSC boundary. In *S. arvensis*, *trnN* and *trnH* were replaced by *BOP3*, and the sequences near this location were virtually identical; this might be explained by the different annotations. With the exception of *S. arvensis* and *S. alba*, there was one more copy of *ycf1* at the LSC/IRb boundary (Figure 6).

### 2.5. Evolution of the Protein-Coding Genes

**Figure 7:**
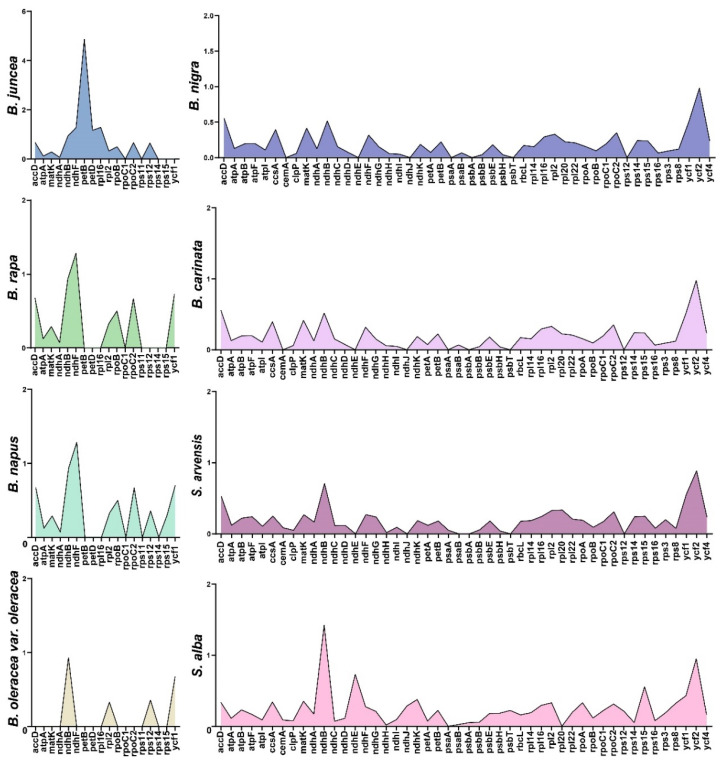
The Ka/Ks ratio of protein-coding genes of ten chloroplast genomes.

Using the SJCT genome as a reference, the Ka/Ks ratio of the FZHH genome was invalid because their Ks values were 0. Genes were considered in the Ka/Ks analysis only when the Ka/Ks ratio was valid. The differences were the lowest between Chinese kale and *B. oleracea* var. *oleracea*. Overall, 13 genes in *B. juncea*, 10 genes in *B. rapa*, 12 genes in *B. napus*, 4 genes in *B. oleracea* var. *oleracea*, 46 genes in *S. alba*, and 41 genes in *B. nigra*, *B. carinata*, and *S. arvensis* had valid Ka/Ks ratios. Some of these genes had Ka/Ks ratios above 1: *ndhF*, *petB*, *petD*, and *rpl16* in *B. juncea*; *ndhF* in *B. rapa* and *B. napus*; and *ndhB* in *S. alba* (Figure 7).

### 2.6. Phylogenetic Analysis

**Figure 8:**
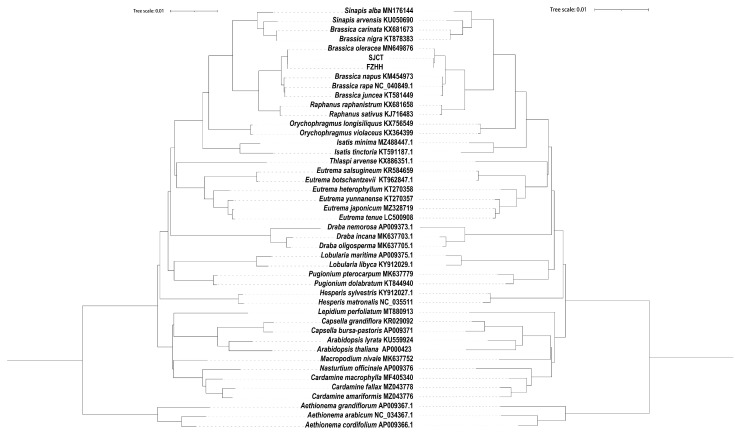
The ML (maximum likelihood) based on 45 complete chloroplast genomes nucleotide sequences (**left**) and protein sequences (**right**).

The topologies of the phylogenies constructed based on nucleotide and protein sequences were similar. *Brassica* was divided into two monophyletic clades. One clade was most closely related to *S. alba*. *S. arvensis* was most closely related to *B. carinata* rather than to *S. alba*. Another clade was closely related to *Raphanus*, and the two Chinese kale varieties were closely related to *B. oleracea* var. *oleracea*. *B. napus*, *B. rapa*, and *B. juncea* were most closely related in both trees (Figure 8).

## 3. Discussion

The height and blade profile of *B. oleracea* var. *alboglabra* are affected by both environmental stimuli and genetic background. SJCT can be easily distinguished from FZHH by its white flower. Although there are some differences among the varieties aside from flower color, these differences can be easily masked by the effect of the environment during the breeding process. Whether white-flower and yellow-flower Chinese kale share the same origin has not yet been established. In other words, the flower color is the standard used to identify variants, but differences in flower color do not necessarily reflect distant relationships.

The complete chloroplast genomes of both Chinese kale variants, six *Brassica*, and two *Sinapis* were included in the comparative analysis. The Triangle of U describes a theory of the evolutionary relationships among six globally important *Brassica* species [17]. This theory posits that the combination of the *Brassica* genomes of three diploid plants formed three modern tetraploid vegetable and rape cultivars. According to the Triangle of U, *B. nigra*, *B. oleracea*, and *B. rapa* were diploid, and *B. carinata*, *B. juncea*, and *B. napus* were tetraploid. The tetraploid species were cultivated from two diploid species. The relationships between these species have been elucidated using various tools. *B. carinata* has been shown to be derived from interspecific hybridization between the diploid progenitors *B. nigra* and *B. oleracea* [18,19]. *S. alba*, also known as *Brassica hirta*, is a white-flowered or yellow-flowered annual plant in the family Brassicaceae [20]. Therefore, we included two Sinapis species in our comparative analysis in addition to the eight Brassica species.

We completed the sequencing, assembly, and annotation of two chloroplast genomes and identified 88 protein-coding genes and eight rRNA genes in the SJCT and FZHH chloroplast genomes. There were 36 tRNA genes in SJCT and 35 tRNA genes in FZHH, and this difference stemmed from the loss of *trnD*. SSRs are an excellent tool for phylogenetic research [21]. They are especially valuable for studies of intraspecific population genetic variation, evolutionary studies at the interspecific level, and species identification [22]. We identified 148–162 SSRs in the 10 chloroplast genomes; the highest number was observed in *B. napus*, and the lowest number was observed in *S. alba*. The number of SSRs in Chinese kale were 151 (SJCT) and 150 (FZHH). In all the chloroplast genomes, there were 11–14 forward repeats (except *B. rapa*, with the maximum number of 20), 1–3 reverse repeats (except *B. rapa*, with the maximum number of 11), and 6–22 reverse complement repeats. Most studies suggest that the chloroplast SSRs were mainly distributed in the non-coding regions; we observed the same in the chloroplast genomes of both SJCT and FZHH.

DNA barcoding can be a useful tool for species identification [23], as it permits the rapid and accurate identification of species via short standard DNA fragments. The DNA barcode of *matK* and *rbcL* was adequate in terms of sequence quality, but these sequences are finite at the species level [24]. Five candidate DNA barcodes for identification (*psbA*, *matk*-*rps16*, *rpl32*, *rpl32*-*trnL*, and *ycf1*) were obtained by a comparison of the nucleotide variability (Pi) between 10 species of Brassicaceae, which is consistent with the results of the mVISTA analysis.

The expansion of IR and the variable SSC regions is considered the main mechanism underlying the length variation in angiosperm chloroplast genomes [25,26]. Generally, the genes at the LSC/IRb and SSC/IRa boundaries were *rps19* and *ycf1*, respectively. The *ycf1* gene of *B. juncea* was lost. These results are consistent with the findings in *Brassica oleracea* var. *italic* [27] and *S. alba* [28].

Synonymous (Ks) and non-synonymous (Ka) nucleotide substitution patterns are important for inferring the evolution of genes, as these metrics are used for assessing selective pressures on protein-coding genes [29]. A Ka/Ks analysis suggested that selection for *rpl2* and *rps12* in both Chinese kale varieties was weak, and this was consistent with the analysis of *ycf1*, *ycf2*, and *rps12*. Protein-coding genes generally experience purifying selection [30,31]. A Ka/Ks analysis of the 10 chloroplast genomes using SJCT as a reference showed that the Ka/Ks ratio of *petB* in *B. juncea* was greater than 1, indicating positive selection. The gene *petB* is a photosynthetic system gene that encodes the protein cytochrome b6, which forms a part of the cytochrome b6f complex (Cytb6f). In the electron transport chain, Cytb6f functions as an electron hub between photosystem II (PSII) and photosystem I (PSI) [32] and plays an important role in photosynthesis. In *B. rapa* and *B. napus*, the Ka/Ks values of *ndhF* were both greater than 1, indicating positive selection. The gene *ndhF* is associated with NADH dehydrogenase [30].

Phylogenetic trees are generally built to determine the genetic relationships between species. A phylogenetic tree based on nucleotide and amino acid sequences revealed that *B. napus*, *B. rapa*, *B. juncea*, and *B. oleracea* var. *oleracea* were clustered into one subgroup and *B. carinata* and *B. nigra* were clustered into another subgroup. These findings are consistent with the results of a previous study showing that *Brassica* coenospecies could be divided into the *Brassica* and *Sinapis* lineages [33]. The results also show that the species most closely related to *B. oleracea* var. *oleracea* is *B. napus*, and this is inconsistent with the results of previous research suggesting that *B. rapa* is the species most closely related to *B. oleracea* var. *oleracea* [34]. This difference might stem from the different genetic modes of the plastid genome and nuclear genome. The same gene in different populations might be subject to convergent selection; in light of the genetic conservatism of the chloroplast genome, *B. nigra* might be the maternal parent of *B. carinata* rather than *B. oleracea* var. *oleracea*, according to the phylogenetic tree of 45 chloroplast genomes, including the chloroplast genomes of the two Chinese kale varieties.

## 4. Materials and Methods

### 4.1. Plant Material and DNA Extraction and Sequencing

Fresh leaves of white flower Chinese kale, *Brassica oleracea* var. *alboglabra* cv. Sijicutiao (SJCT) and yellow flower Chinese kale, *Brassica oleracea* var. *alboglabra* cv. Fuzhouhuanghua (FZHH) were collected from the College of Horticulture, Sichuan Agricultural University. The total genomic DNA was extracted from the leaves according to a modified CTAB method [35]. Fifteen pairs of universal primer were used for PCR amplifications (Supplemental Table S5) [36]. The paired reads with 300 bp insert size sequencing were performed with an Illumina Hiseq X Ten platform by Shenzhen BGI Genomics Co., Ltd. (Shenzhen, China) [37]. Raw data filtering was used to obtain clean reads (2 GB) by Trimmomatic v0.22 [38].

### 4.2. Gene Assembly and Annotation

Pilon (https://github.com/broadinstitute/pilon/releases (accessed on 25 May 2022)) was utilized to correct the spliced sequence [39]. The chloroplast genomes were assembled by SPAdes v.3.11.0 [40] using the chloroplast genome of *B. oleracea* var. *oleracea* (NC_041167.1) as a reference. The complete chloroplast genome sequences were annotated using Chloroplast Genome Annotation, Visualization, Analysis, and GenBank Submission (CPGAVAS) (http://47.96.249.172:16019/analyzer/home (accessed on 15 January 2023)) [41]. The organisms were named *Brassica oleracea* var. *alboglabra* cv. Sijicutiao and *Brassica oleracea* var. *alboglabra* cv. Fuzhouhuanghua, and the sequences were submitted to GenBank (Accession number OR063915 and OR063916). The circular genome map was drawn using Chloroplot (https://irscope.shinyapps.io/Chloroplot/ (accessed on 15 January 2023)) [42].

### 4.3. Sequence Analysis and Statistics

The relative synonymous codon usage (RSCU) analysis was produced using CodonW (http://codonw.sourceforge.net/ (accessed on 18 February 2023)). REPuter (https://bibiserv.cebitec.uni-bielefeld.de/reputer/ (accessed on 19 February 2023)) was used to analyze the repeat sequences from 30 bp to 100 bp, and the hamming distance was set to 3. A simple sequence repeat (SSR) analysis was conducted using MIcroSAtellite (MISA) [43], and the minimum number of repeats was set to 8, 5, 3, 3, and 3 for mononucleotides, dinucleotides, trinucleotides, tetranucleotides, pentanucleotides, and hexanucleotides, respectively [44].

### 4.4. Genome Comparison

Two Chinese kale varieties, six species of the Triangle of U (*B. oleracea*, *B. rapa*, *B. napus*, *B. juncea*, *B. nigra*, and *B. carinata*), and two species of *Sinapis* (*Sinapis alba* and *S. arvensis*), which is considered the genus most closely related to *Brassica*, were included in the comparative genome analysis.

The annotations were compared by mVISTA in the Shuffle-LAGAN mode using SJCT as a reference [45]. The whole chloroplast genome sequences were aligned in Geneious 9.0.2 (https://www.geneious.com (accessed on 24 March 2023)) with Mauve 2.3.1. The contraction and expansion of the IR boundaries were detected and visualized between four main regions (LSC/IRb/SSC/IRa) of 10 chloroplast genome sequences using IRSCOPE (https://irscope.shinyapps.io/irapp/ (accessed on 24 March 2023)). The nucleotide variability was calculated using DnaSP v5.10 [46]. The Ka/Ks values were calculated with KaKs_calculator 2.0, using SJCT as a reference [47].

### 4.5. Phylogenetic Analysis

The protein-coding sequences and the nucleotide sequences of 45 chloroplast genomes, including SJCT and FZHH, were used to conduct the phylogenetic analysis. Chloroplast genomes of 43 other species were downloaded from NCBI, including three chloroplast genome sequences used as outgroup species (Supplemental Table S6). The chloroplast genome sequences were aligned using MAFFT v7 [48], and conserved sequences were extracted using HomBlocks [49] with the G-blocks. The phylogenetic trees were constructed using IQ-tree [50], and a maximum likelihood (ML) analysis was performed with the best-fit model JTT+F+R3 for the protein-coding sequences (TVM+F+R3 for the nucleotide sequences).

## 5. Conclusions

In this study, the complete chloroplast genomes of two Chinese kale varieties were sequenced and analyzed for the first time. They both contained 88 protein-coding genes and eight rRNA genes; there were 36 and 35 tRNA genes in SJCT and FZHH, respectively. The analysis of the 10 chloroplast genomes of Brassicaceae revealed that their RSCU values were similar. Five highly variable regions of DNA barcodes were detected. The structures of the chloroplast genomes were conserved. Using Chinese kale as a reference, we found that the lowest number of differential genes was observed in *B. oleracea* var. *oleracea* and natural selection has shaped the evolution of these genes. These results suggest that both white-flower and yellow-flower Chinese kale are derived from one common monophyletic origin, and these two Chinese kale varieties likely possessed different colors late in the artificial selection process.

## Data Availability

All data supporting the findings of this study are available within the paper and within its Supplementary Materials published online.

## Supplementary Materials

The supporting information can be downloaded at: https://www.mdpi.com/article/10.3390/ijms241210287/s1.
