# Supplementary material for: Sequencing and Analysis of Complete Chloroplast Genomes Provide Insight into the Evolution and Phylogeny of Chinese Kale (Brassica oleracea var. alboglabra)
Source: Int J Mol Sci. 2023 Jun 17;24(12):10287. doi: 10.3390/ijms241210287 (PMC10299174; doi:10.3390/ijms241210287)
Supplement: Supplementary file 1 [file ijms-24-10287-s001.zip › Supplemental Table S1 List of gene function in the chloroplast genome of SJCT.pdf]

Supplemental Table S1 List of gene function in the chloroplast genome of SJCT

| Categoty | Gene group | Gene name |
|---|---|---|
| Photosynthesis | Subunits of photosystem I | *psaA,psaB,psaC,psaI,psaJ* |
| | Subunits of photosystem II | *psbA,psbB,psbC,psbD,psbE,psbF,psbH,psbI,psbJ,psbK,psbL,psbM,psbN,psbT,psbZ* |
| | Subunits of NADH dehydrogenase | *ndhA*,ndhB*(2),ndhC,ndhD,ndhE,ndhF,ndhG,ndhH,ndhI,ndhJ,ndhK* |
| | Subunits of cytochrome b/f complex | *petA,petB*,petD*,petG,petL,petN* |
| | Subunits of ATP synthase | *atpA,atpB,atpE,atpF*,atpH,atpI* |
| | Large subunit of rubisco | *rbcL* |
| | Subunits photochlorophyllide reductase | - |
| Self-replication | Proteins of large ribosomal subunit | *rpl14,rpl16*,rpl2*(2),rpl20,rpl22,rpl23(2),rpl32,rpl33,rpl36* |
| | Proteins of small ribosomal subunit | *rps11,rps12**(2),rps14,rps15,rps16*,rps18,rps19,rps2,rps3,rps4,rps7(2),rps8* |
| | Subunits of RNA polymerase | *rpoA,rpoB,rpoC1*,rpoC2* |
| | Ribosomal RNAs | *rrn16S(2),rrn23S(2),rrn4.5S(2),rrn5S(2)* |
| | Transfer RNAs | *trnA-UGC*(2),trnC-GCA,trnD-GUC,trnE-UUC,trnF-GAA,trnG-GCC,trnG-UCC*,trnH-GUG, trnI-CAU(2),trnI-GAU*(2),trnK-UUU*,trnL-CAA(2),trnL-UAA*,trnL-UAG,trnM-CAU(2), trnN-GUU(2),trnP-UGG,trnQ-UUG,trnR,trnR-UCU,trnS-GCU,trnS-GGA,trnS-UGA,trnT-GGU, trnT-UGU,trnV-GAC(2),trnV-UAC*,trnW-CCA,trnY-GUA* |
| Other genes | Maturase | *matK* |
| | Protease | *clpP** |
| | Envelope membrane protein | *cemA* |
| | Acetyl-CoA carboxylase | *accD* |
| | c-type cytochrome synthesis gene | *ccsA* |
| | Translation initiation factor | - |
| | other | - |
| Genes of unknown function | Conserved hypothetical chloroplast ORF | *ycf1(2),ycf15(2),ycf2(2),ycf3**,ycf4* |

Notes:Gene*:Gene with one introns;Gene**:Gene with two introns;#Gene:Pseudo gene;Gene(2):Number of copies of multi-copy genes;
