# Supplementary material for: Sequencing and Analysis of Complete Chloroplast Genomes Provide Insight into the Evolution and Phylogeny of Chinese Kale (Brassica oleracea var. alboglabra)
Source: Int J Mol Sci. 2023 Jun 17;24(12):10287. doi: 10.3390/ijms241210287 (PMC10299174; doi:10.3390/ijms241210287)
Supplement: Supplementary file 1 [file ijms-24-10287-s001.zip › Supplemental Table S2 List of gene function in the chloroplast genome of FZHH.pdf]

Supplemental Table S2 List of gene function in the chloroplast genome of FZHH

Categoty	Gene group	Gene name
Photosynthesis	Subunits of photosystem I	*psaA,psaB,psaC,psaI,psaJ*
	Subunits of photosystem II	*psbA,psbB,psbC,psbD,psbE,psbF,psbH,psbI,psbJ,psbK,psbL,psbM,psbN,psbT,psbZ*
	Subunits of NADH dehydrogenase	*ndhA*,ndhB*(2),ndhC,ndhD,ndhE,ndhF,ndhG,ndhH,ndhI,ndhJ,ndhK*
	Subunits of cytochrome b/f complex	*petA,petB*,petD*,petG,petL,petN*
	Subunits of ATP synthase	*atpA,atpB,atpE,atpF*,atpH,atpI*
	Large subunit of rubisco	*rbcL*
	Subunits photochlorophyllide reductase	-
Self-replication	Proteins of large ribosomal subunit	*rpl14,rpl16*,rpl2*(2),rpl20,rpl22,rpl23(2),rpl32,rpl33,rpl36*
	Proteins of small ribosomal subunit	*rps11,rps12**(2),rps14,rps15,rps16*,rps18,rps19,rps2,rps3,rps4,rps7(2),rps8*
	Subunits of RNA polymerase	*rpoA,rpoB,rpoC1*,rpoC2*
	Ribosomal RNAs	*rrn16S(2),rrn23S(2),rrn4.5S(2),rrn5S(2)*
	Transfer RNAs	*trnA-UGC*(2),trnC-GCA,trnE-UUC,trnF-GAA,trnG-GCC,trnG-UCC*,trnH-GUG,trnI-CAU(2), trnI-GAU*(2),trnL-CAA(2),trnL-UAA*,trnL-UAG,trnM-CAU(2),trnN-GUU(2),trnP-UGG, trnQ-UUG,trnR,trnR-UCU,trnS-GCU,trnS-GGA,trnS-UGA,trnT-GGU,trnT-UGU,trnV-GAC(2), trnV-UAC*,trnW-CCA,trnY-GUA*
Other genes	Maturase	*matK*
	Protease	*clpP**
	Envelope membrane protein	*cemA*
	Acetyl-CoA carboxylase	*accD*
	c-type cytochrome synthesis gene	*ccsA*
	Translation initiation factor	-
	other	-
Genes of unknown function	Conserved hypothetical chloroplast ORF	*ycf1(2),ycf15(2),ycf2(2),ycf3**,ycf4*

Notes:Gene*:Gene with one introns;Gene**:Gene with two introns;#Gene:Pseudo gene;Gene(2):Number of copies of multi-copy genes;
