# Supplementary material for: Sequencing and Analysis of Complete Chloroplast Genomes Provide Insight into the Evolution and Phylogeny of Chinese Kale (Brassica oleracea var. alboglabra)
Source: Int J Mol Sci. 2023 Jun 17;24(12):10287. doi: 10.3390/ijms241210287 (PMC10299174; doi:10.3390/ijms241210287)
Supplement: Supplementary file 1 [file ijms-24-10287-s001.zip › Supplemental Table S4 List of RSCU in ten chloroplast genomes.pdf]

Supplemental Table S4 List of RSCU in ten chloroplast genomes

Symbol	Codon	SJCT	FZHH	*Brassica oleracea*	*Brassica rapa*	*Brassica napus*	*Brassica juncea*	*Brassica nigra*	*Brassica carinata*	*Sinapis arvensis*	*Sinapis alba*
Ter	UAA	1.7625	1.7625	1.7625	1.8375	1.8148	1.7848	1.7625	1.7931	1.7711	1.7711
Ter	UAG	0.75	0.75	0.75	0.7125	0.7407	0.7595	0.75	0.7241	0.759	0.759
Ter	UGA	0.4875	0.4875	0.4875	0.45	0.4444	0.4557	0.4875	0.4828	0.4699	0.4699
Ala	GCA	1.1075	1.1075	1.1075	1.1209	1.1165	1.1082	1.1079	1.1034	1.1185	1.1185
Ala	GCC	0.5714	0.5714	0.5746	0.5669	0.5692	0.577	0.5797	0.5838	0.6093	0.6093
Ala	GCG	0.4302	0.4302	0.427	0.4259	0.4253	0.4262	0.4444	0.4491	0.4297	0.4297
Ala	GCU	1.8909	1.8909	1.8909	1.8863	1.889	1.8885	1.868	1.8637	1.8425	1.8425
Cys	UGC	0.48	0.48	0.4727	0.4689	0.4648	0.4585	0.4762	0.4894	0.4777	0.4777
Cys	UGU	1.52	1.52	1.5273	1.5311	1.5352	1.5415	1.5238	1.5106	1.5223	1.5223
Asp	GAC	0.3959	0.3959	0.3945	0.3959	0.3924	0.399	0.3862	0.3904	0.384	0.384
Asp	GAU	1.6041	1.6041	1.6055	1.6041	1.6076	1.601	1.6138	1.6096	1.616	1.616
Glu	GAA	1.5499	1.5499	1.5497	1.5497	1.5513	1.5311	1.5483	1.5474	1.5149	1.5149
Glu	GAG	0.4501	0.4501	0.4503	0.4503	0.4487	0.4689	0.4517	0.4526	0.4851	0.4851
Phe	UUC	0.5993	0.5993	0.5963	0.5948	0.6053	0.6139	0.6034	0.6041	0.6388	0.6388
Phe	UUU	1.4007	1.4007	1.4037	1.4052	1.3947	1.3861	1.3966	1.3959	1.3612	1.3612
Gly	GGA	1.6128	1.6128	1.6143	1.6178	1.6343	1.6063	1.6117	1.6096	1.6534	1.6534
Gly	GGC	0.3885	0.3885	0.3921	0.3924	0.3881	0.3937	0.3921	0.3904	0.3869	0.3869
Gly	GGG	0.6594	0.6594	0.6544	0.6522	0.6468	0.6509	0.6518	0.6565	0.6586	0.6586
Gly	GGU	1.3393	1.3393	1.3393	1.3376	1.3308	1.3491	1.3444	1.3435	1.3011	1.3011
His	CAC	0.4942	0.4942	0.4962	0.5	0.4962	0.5071	0.5153	0.5245	0.4958	0.4958
His	CAU	1.5058	1.5058	1.5038	1.5	1.5038	1.4929	1.4847	1.4755	1.5042	1.5042
Ile	AUA	0.9429	0.9429	0.9427	0.9466	0.9452	0.9263	0.9526	0.9522	0.962	0.962
Ile	AUC	0.5375	0.5375	0.5408	0.5317	0.5381	0.5525	0.5257	0.5262	0.5427	0.5427
Ile	AUU	1.5195	1.5195	1.5164	1.5217	1.5167	1.5211	1.5217	1.5217	1.4954	1.4954
Lys	AAA	1.5639	1.5655	1.5646	1.5607	1.5608	1.5193	1.56	1.5576	1.5291	1.5291
Lys	AAG	0.4361	0.4345	0.4354	0.4393	0.4392	0.4807	0.44	0.4424	0.4709	0.4709
Leu	CUA	0.819	0.819	0.8163	0.8023	0.8198	0.7856	0.8185	0.8158	0.8322	0.8322
Leu	CUC	0.3801	0.3801	0.3764	0.3767	0.3873	0.3784	0.3613	0.365	0.3779	0.3779
Leu	CUG	0.3482	0.3482	0.347	0.3473	0.3493	0.3471	0.349	0.3503	0.356	0.356
Leu	CUU	1.2309	1.2309	1.2367	1.2303	1.238	1.2371	1.2216	1.225	1.2559	1.2559
Leu	UUA	2.1406	2.1406	2.1434	2.1623	2.141	2.1479	2.1508	2.1462	2.0379	2.0379
Leu	UUG	1.0813	1.0813	1.0802	1.0811	1.0646	1.104	1.0987	1.0976	1.1402	1.1402
Met	AUG	1	1	1	1	1	1	1	1	1	1
Asn	AAC	0.4496	0.4496	0.4502	0.4452	0.4497	0.4447	0.4419	0.4462	0.4548	0.4548
Asn	AAU	1.5504	1.5504	1.5498	1.5548	1.5503	1.5553	1.5581	1.5538	1.5452	1.5452
Pro	CCA	1.1578	1.1578	1.1589	1.1577	1.161	1.1435	1.1404	1.1404	1.1134	1.1134
Pro	CCC	0.7138	0.7138	0.7092	0.7041	0.6949	0.7107	0.7127	0.7203	0.7436	0.7436
Pro	CCG	0.5354	0.5354	0.5319	0.54	0.5339	0.5376	0.5227	0.523	0.5415	0.5415
Pro	CCU	1.593	1.593	1.6	1.5983	1.6102	1.6082	1.6242	1.6163	1.6015	1.6015
Gln	CAA	1.5674	1.5674	1.568	1.568	1.5683	1.5582	1.5774	1.576	1.5565	1.5565
Gln	CAG	0.4326	0.4326	0.432	0.432	0.4317	0.4418	0.4226	0.424	0.4435	0.4435
Arg	AGA	1.7666	1.7666	1.7684	1.7562	1.7558	1.706	1.7713	1.7697	1.8008	1.8008
Arg	AGG	0.5738	0.5738	0.5729	0.5779	0.5719	0.5831	0.574	0.584	0.6131	0.6131
Arg	CGA	1.3554	1.3554	1.3624	1.3589	1.376	1.359	1.3632	1.3551	1.365	1.365
Arg	CGC	0.4337	0.4337	0.4331	0.4379	0.4334	0.453	0.4305	0.4279	0.4049	0.4049
Arg	CGG	0.4654	0.4654	0.4647	0.465	0.4691	0.4867	0.4888	0.4948	0.482	0.482
Arg	CGU	1.4051	1.4051	1.3985	1.4041	1.3939	1.412	1.3722	1.3685	1.3342	1.3342
Ser	AGC	0.3507	0.3507	0.3497	0.3494	0.3659	0.3538	0.3538	0.3546	0.3677	0.3677
Ser	AGU	1.2554	1.2554	1.2587	1.2545	1.2332	1.2685	1.2399	1.248	1.2108	1.2108
Ser	UCA	1.2168	1.2168	1.2168	1.2021	1.2095	1.1669	1.1944	1.1958	1.2018	1.2018
Ser	UCC	0.8311	0.8311	0.8287	0.8317	0.8402	0.8582	0.8476	0.8413	0.8759	0.8759
Ser	UCG	0.547	0.547	0.549	0.5556	0.5488	0.5571	0.5534	0.5597	0.5919	0.5919
Ser	UCU	1.7989	1.7989	1.7972	1.8066	1.8024	1.7955	1.8109	1.8007	1.7519	1.7519
Thr	ACA	1.2446	1.2446	1.245	1.2338	1.2194	1.2186	1.2562	1.2592	1.2517	1.2517
Thr	ACC	0.7155	0.7155	0.7219	0.7197	0.7226	0.7168	0.7074	0.713	0.6931	0.6931
Thr	ACG	0.396	0.396	0.3974	0.408	0.4097	0.405	0.4066	0.4154	0.4302	0.4302
Thr	ACU	1.6439	1.6439	1.6358	1.6385	1.6484	1.6595	1.6298	1.6124	1.6251	1.6251
Val	GUA	1.454	1.454	1.4457	1.4505	1.4545	1.4657	1.4671	1.4636	1.4652	1.4652
Val	GUC	0.4941	0.4941	0.4934	0.4961	0.4931	0.4864	0.4859	0.491	0.4903	0.4903
Val	GUG	0.5413	0.5413	0.5464	0.54	0.5516	0.5558	0.5361	0.5379	0.5563	0.5563
Val	GUU	1.5106	1.5106	1.5144	1.5133	1.5008	1.4921	1.511	1.5074	1.4882	1.4882
Trp	UGG	1	1	1	1	1	1	1	1	1	1
Tyr	UAC	0.3702	0.3702	0.3693	0.3662	0.3699	0.3693	0.3638	0.3628	0.3731	0.3731
Tyr	UAU	1.6298	1.6298	1.6307	1.6338	1.6301	1.6307	1.6362	1.6372	1.6269	1.6269
