# Supplementary material for: Sequencing and Analysis of Complete Chloroplast Genomes Provide Insight into the Evolution and Phylogeny of Chinese Kale (Brassica oleracea var. alboglabra)
Source: Int J Mol Sci. 2023 Jun 17;24(12):10287. doi: 10.3390/ijms241210287 (PMC10299174; doi:10.3390/ijms241210287)
Supplement: Supplementary file 1 [file ijms-24-10287-s001.zip › Supplemental Table S5 List of primers.pdf]

Supplemental Table S5 List of primers

Primer ID	Primer sequence (5′ to 3′)
CPUP_1F_trnK-UUU	GCACTTAAAAGCCGARTACTCTACCR
CPUP_1R_trnR-UCU	CAAAGGTTTAGAAGACCTMTGTCCTATCCA
CPUP_2F_trnR-UCU	CCATTGTCTAATGGATAGGACAKAGGTC
CPUP_2R_rpoC2	AGGACAAATGATYGATTTACCYATTCAAAG
CPUP_3F_rpoC2	TRCTTTGAATRGGTAAATCRATCATTTGT
CPUP_3R_trnD-GUC	CTGTCAAGGCGGAAGCTRCGGG
CPUP_4F_trnD-GUC	GAACCCGYAGCTTCCGCCTTGAC
CPUP_4R_psaA	TTAAAAGTTGCTCCTGCTACTCAGCC
CPUP_5F_psaA	YAAGGCTCTMGGCTGAGTAGCAGGAG
CPUP_5R_trnF-GAA	ACCAGATTTGAACTGGTGACACGAGGA
CPUP_6F_trnF-GAA	ACTGAAAATCCTCGTGTCACCAGTTCA
CPUP_6R_rbcL	GTGBATACCATGATTYYTCTGTCTATCA
CPUP_7F_rbcL	GYGCAATGCAYGCHGTTATTGATAGA
CPUP_7R_trnW-CCA	AGGTTCAAATCCTAYAGAGCGTGAT
CPUP_8F_trnW-CCA	ATCACGCTCTRTAGGATTTGAACC
CPUP_8R_petD	TCAATAGTTCAAGTCACACACTCCCA
CPUP_9F_petD	TGGGAGTGTGTGACTTGAACTATTGATT
CPUP_9R_trnI-CAU	ATCCATGGCTGAATGGTTAAAGCGCC
CPUP_10F_trnI-CAU	TTYGCCAATTATGAGTTGGGCGCTTT
CPUP_10R_rps7	AGGAAAAGCACTTGCCATTCGTTGGT
CPUP_11F_rps7	CCATATTTCGACCCGGACGTTTTCGG
CPUP_11R_rrn4.5	GATGCCTCAGCTGCATACATCACTGC
CPUP_12F_rrn4.5	ATGCAGCTGAGGCATCCTAACAGACC
CPUP_12R_trnL-UAG	TGCTTCCTAAGAGCAGCGTGTCTACC
CPUP_13F_trnL-UAG	GGTAGACACGCTGCTCTTAGGAAG
CPUP_13R_rps15	CACCAATAAGATACGAAGACTTACTTCACA
CPUP_14F_rps15	TGTGCAATTCCAAATGTGAAGTAAGTCT
CPUP_14R_trnR-ACG	GGTTCGAATCCCTCCTCGCCCA
CPUP_15F_trnI-CAU	ATCCATGGCTGAATGGTTAAAGCGCC
CPUP_15R_trnK-UUU	CTCAACGGTAGAGTACTCGGCTT
CPUP_15-1F_trnI-CAU	TCAATTCCTRCTGGATGCACGC
CPUP_15_1_0F_rps7	CACAAGGAAAAGCACTTGCCAT
CPUP_15-1_1F_trnI-CAU	AGCATCCATGGCTGAATGGTTA
CPUP_15-1R_rps16	CGATGTGGTAGAAAGCAACGTG
rRNA_2F	TAAGCCATGCATGTGTAAGTATGAAC
rRNA_2R	CGTATTTAAGTCGTCTGCAAAGGATT
