# Supplementary material for: Sequencing and Analysis of Complete Chloroplast Genomes Provide Insight into the Evolution and Phylogeny of Chinese Kale (Brassica oleracea var. alboglabra)
Source: Int J Mol Sci. 2023 Jun 17;24(12):10287. doi: 10.3390/ijms241210287 (PMC10299174; doi:10.3390/ijms241210287)
Supplement: Supplementary file 1 [file ijms-24-10287-s001.zip › Supplemental Table S6 List of accession numbers in Genbank.pdf]

Supplemental Table S6 List of accession numbers in Genbank

| Species | Accession umbers in Genbank |
|---|---|
| *Aethionema arabicum* | NC_034367.1 |
| *Aethionema cordifolium* | AP009366.1 |
| *Aethionema grandiflorum* | AP009367.1 |
| *Arabidopsis lyrata* | KU559924.1 |
| *Arabidopsis thaliana* | AP000423.1 |
| *B. oleracea* var. *alboglabra* cv. Sijicutiao | OR063915 |
| *B. oleracea* var. *alboglabra* cv. Fuzhouhuanghua | OR063916 |
| *B. oleracea* var. *oleracea* | MN649876.1 |
| *Brassica carinata* | KX681673.1 |
| *Brassica juncea* | KT581449.1 |
| *Brassica napus* | KM454973.1 |
| *Brassica nigra* | KT878383.1 |
| *Brassica rapa* | NC_040849.1 |
| *Capsella bursa-pastoris* | AP009371.1 |
| *Capsella grandiflora* | KR029092.1 |
| *Cardamine amariformis* | MZ043776.1 |
| *Cardamine fallax* | MZ043778.1 |
| *Cardamine macrophylla* | MF405340.1 |
| *Draba incana* | MK637703.1 |
| *Draba nemorosa* | AP009373.1 |
| *Draba oligosperma* | MK637705.1 |
| *Eutrema heterophyllum* | KT270358.1 |
| *Eutrema japonicum* | MZ328719 |
| *Eutrema salsugineum* | MK637715.1 |
| *Eutrema tenue* | LC500908.1 |
| *Eutrema yunnanense* | KT270357.1 |
| *Hesperis matronalis* | NC_035511.1 |
| *Hesperis sylvestris* | KY912027.1 |
| *Isatis minima* | MZ488447.1 |
| *Isatis tinctoria* | KT591187.1 |
| *Lepidium perfoliatum* | MT880913.1 |
| *Lobularia libyca* | KY912029.1 |
| *Lobularia maritima* | AP009375.1 |
| *Macropodium nivale* | MK637752.1 |
| *Nasturtium officinale* | AP009376.1 |
| *Orychophragmus longisiliquus* | KX756549 |
| *Orychophragmus violaceus* | KX364399.1 |
| *Pugionium dolabratum* | KT844940.1 |
| *Pugionium pterocarpum* | MK637779.1 |
| *Raphanus raphanistrum* | KX681658.1 |
| *Raphanus sativus* | KJ716483.1 |
| *Schrenkiella parvula* | KR584659.1 |
| *Sinapis alba* | MN176144.1 |
| *Sinapis arvensis* | KU050690.1 |
| *Thlaspi arvense* | KX886351.1 |
