# Supplementary figures and images for: Sequencing and Analysis of Complete Chloroplast Genomes Provide Insight into the Evolution and Phylogeny of Chinese Kale (Brassica oleracea var. alboglabra)
Source: Int J Mol Sci. 2023 Jun 17;24(12):10287. doi: 10.3390/ijms241210287 (PMC10299174; doi:10.3390/ijms241210287)

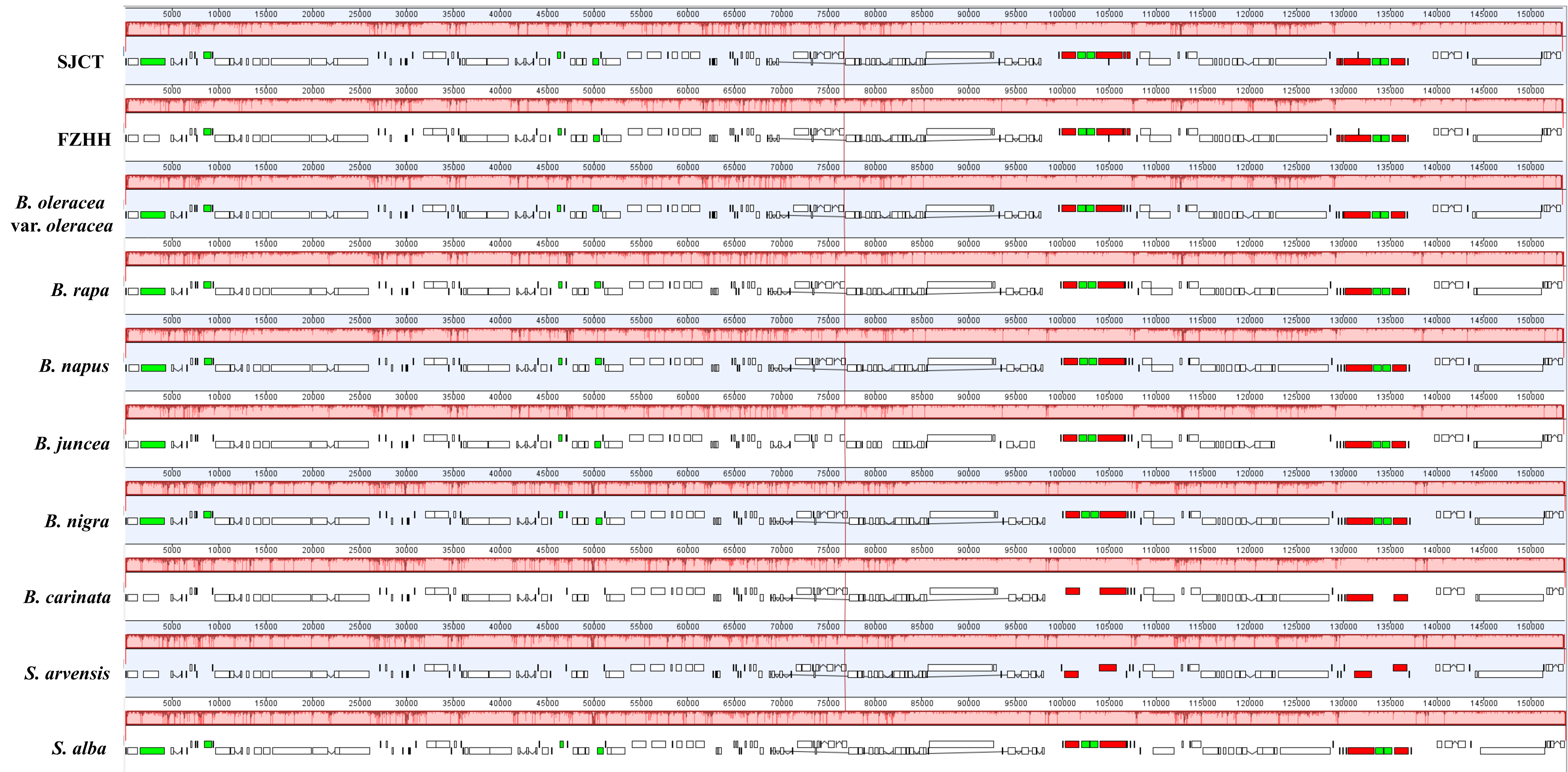

**Supplementary Figure S1:** Synteny analyses for ten species in chloroplast genomes.

Supplement: Supplementary file 1 [file ijms-24-10287-s001.zip › Supplemental Figure S1.pdf]
